# Intercompartmental Piecewise Gene Transfer

**DOI:** 10.3390/genes8100260

**Published:** 2017-10-06

**Authors:** Przemyslaw Szafranski

**Affiliations:** Department of Molecular and Human Genetics, Baylor College of Medicine, One Baylor Plaza, Houston, TX 77030, USA; pszafran@bcm.edu; Tel.: +1-713-798-5375

**Keywords:** endosymbiotic gene transfer, mobile elements, functional gene fragmentation, mitochondrial DNA

## Abstract

Gene relocation from the residual genomes of organelles to the nuclear genome still continues, although as a scaled down evolutionary phenomenon, limited in occurrence mostly to protists (*sensu lato*) and land plants. During this process, the structural integrity of transferred genes is usually preserved. However, the relocation of mitochondrial genes that code for respiratory chain and ribosomal proteins is sometimes associated with their fragmentation into two complementary genes. Herein, this review compiles cases of piecewise gene transfer from the mitochondria to the nucleus, and discusses hypothesized mechanistic links between the fission and relocation of those genes.

## 1. Introduction

Intercompartmental, evolutionary gene transfer from the greatly reduced genomes of mitochondria (mtDNA), plastids and nucleomorphs to the nuclear genome is, according to the endosymbiotic theory of organelle origin, a continuation of horizontal (or lateral) relocation of genes from pre-organellar endosymbionts of evolving eukaryotic cells [1,2,3,4,5,6]. It has been proposed that during the early stages of mitochondrial or chloroplast evolution, there was a massive horizontal transfer of genes from symbiotic α-proteobacteria or cyanobacteria to an archaeon presumably related to the Lokiarchaeota, followed by lineage-specific integration, differentiation, or loss of relocated genes, with subsequent transfers being highly discontinuous [7]. A fraction of protein-coding genes that have been relocated acquired mitochondrion or plastid targeting sequences, allowing their products to localize back to those organelles.

Although the endosymbiotic hypothesis, particularly the extent of organelle precursor contribution to eukaryotic genomes and proteomes, continues to be disputed by some [8], gene flow between cellular compartments, especially from organelles to the nucleus, is a well-documented phenomenon that has been verified experimentally [5,6,9]. Cases of evolutionarily relatively recent, functional organelle gene translocations to the nuclear genome have been described in protists (*sensu lato*) and land plants but not, for instance, in animals or fungi [1,2,3,4,5]. However, integration of organelle or endosymbiont DNA fragments to the nuclear genome (or sometimes transfer of the entire organelle DNA), occurs in almost all eukaryotic organisms, although it usually results in the generation of nuclear pseudogenes of organellar or endosymbiont genes [2,3,5,10,11,12]. Such a DNA transfer may also contribute to the development of novel exonic sequences in existing nuclear genes [9,13]. Approximately 10% of mitochondrion and plastid-derived nuclear genome insertions (NUMTs and NUPTs respectively) occurred within protein-coding exons [9], and the functionality of the resulting mosaic genes has been demonstrated for a subset of those insertions [9,13]. In contrast to organelle gene transfer to the nucleus, identified cases of gene transfer between mitochondria and chloroplasts are scarcer, and the significance of nuclear gene relocation to mitochondria or chloroplasts is still debated [2,5].

The typically short life span of organellar homologs of genes transferred to the nuclear genome argues against the neutrality of intercompartmental gene relocations. The localization to the nucleus of genes for organelle proteins and ribosomal/transfer RNAs (r/tRNAs) may have a selective advantage because of the deleterious effects of Muller’s ratchet (accumulation of mutations in asexually propagated organelle DNA) [14,15,16], especially when the organelle genome has a higher nucleotide substitution rate compared to the nuclear genome. Likewise, it might be advantageous in situations when high levels of free radicals are a factor [17], as is the case for increased nucleotide substitution rate or instability of organelle genome architecture. Gene transfer to the nucleus also allows for size reduction of the organelle DNA, which is a trend generally followed by endosymbionts, and may be conditioned by a tendency towards the reduction of energetic costs of DNA maintenance and expression [18]. Lastly, selection asymmetry may favor the movement of mitochondrial genes to the nucleus in cases of uniparentally inherited mitochondria [19].

**Figure 1:**
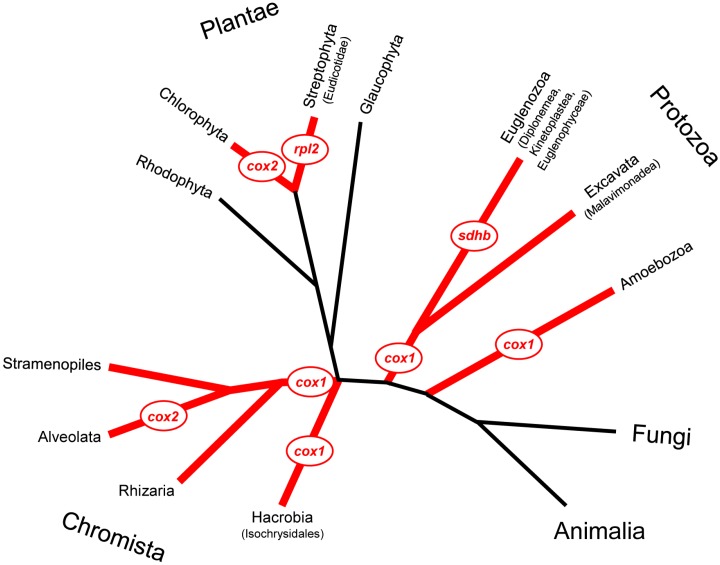
Piecewise mitochondrial gene transfer to the nucleus across phylogeny. Major eukaryotic groups harbouring taxa featuring one or two fragmented genes that have been at least partially relocated from mitochondrial DNA (mtDNA) to the nuclear genome are marked in red. The simplified tree topology and taxonomy are based on [52] and [36], respectively.

It is often impossible to assess the integrity of organelle genes that were relocated early in eukaryotic evolution because of advanced divergence of their sequences. During more recent relocations, most transferred genes appear intact within their new loci. However, several protein-coding genes were relocated to the nuclear genome either incompletely, as a half or shorter part of a gene, or completely but in the form of two non-overlapping gene pieces likely in two independent transfer events (Table 1, Figure 1). There are also known cases of fragmented, yet apparently functional, mitochondrial rRNA genes [20,21], as well as incomplete tRNA genes [22], with some of their essential regions absent from mtDNA, suggesting possible transfer to the nuclear genome and posttranscriptional importation. Genes that become fragmented into derived genes are expressed as polypeptides or r/tRNAs that are thought to non-covalently reassemble into heterodimers (or heteromultimers in some cases of rRNAs), exhibiting activities of an intact protein or RNA [20,21,22,23]. In fact, several research tools based on gene/protein complementation assays, including yeast-two-hybrid, split green fluorescent protein (GFP) [24], luciferase [25], GAL4 [26] or split Cas9 system [27], took advantage of functional gene fission and reassembly of derived gene products, and independently verify functionality of the genes-in-pieces arrangement in general. In terms of evolution, gene fragmentation, when followed by combinatorial fusions, is one of the major ways to develop new molecular structures and activities [28,29]. It also reduces problems associated with folding large proteins, and creates additional opportunities for regulation of the expression and function of the corresponding RNA or protein heteromultimeric complexes [30,31,32].

Here, I review piecewise gene transfers from mitochondria to the nucleus, partial nuclear and organelle gene conversions, and discuss mechanisms of gene transfer in the context of functional gene fission.

## 2. Cases of Piecewise Mitochondrial Gene Relocation to the Nucleus

### 2.1. The Cox2 Gene

#### 2.1.1. Endosymbiotic Transfer of Cox2 in Chlorophyceae (Chlorophyta) and Alveolata

*Cox2* was the first gene reported to be transferred from mtDNA to the nuclear genome in pieces as complementary *cox2a* and *cox2b* genes [33,34,35]. *Cox2* encodes the subunit II of cytochrome c (CytC) oxidase (COX) that mediates the transfer of electrons from CytC to COX subunit I (COXI) during oxidative phosphorylation (OXPHOS). COXII is anchored in the inner mitochondrial membrane by two transmembrane helices. A redox center of COXII, Cu_A_, is a part of the intermembrane space-exposed cupredoxin domain comprising most of the C-terminal half of COXII. In all known cases of fragmented *cox2*, the split occurred in the same, relatively less conserved position within the gene, between regions coding for the transmembrane and cupredoxin domains.

The assembly of an active COXII likely involves interactions in the mitochondrial intermembrane space of COXIIA and COXIIB termini [35,44] and is apparently assisted by other proteins of the respiratory Complex IV.

The split-*cox2* arrangement was originally identified in three genera of chlorophycean algae, *Polytomella*, *Chlamydomonas* and *Scenedesmus* [33,34,35], and in the apicomplexan parasites (Alveolata) such as *Toxoplasma*, *Plasmodium* and *Theileria* [44,45].

It has been proposed that *cox2* underwent fission in a common ancestor of the extant Chlorophyceae, followed by relocation of the *cox2b* to the nucleus. Subsequently, *cox2a* might have been transferred to the nucleus in Chlamydomonadales (e.g., in *Chlamydomonas*, *Polytomella*) but not in Chaetophorales or *Scenedesmus* and other Sphaeropleales [46]. Sequential relocation of *cox2a* and *cox2b* to the nucleus is further supported by localization of these two complementary genes on different chromosomes in, e.g., *Chlamydomonas* [53,54]. In Alveolata, split *cox2* has been later identified also in dinoflagellates *Karlodinium* and *Oxyrrhis* as well as *Perkinsus* [48]. In contrary to the previously suggested horizontal transfer of *cox2a* and *cox2b* from the nucleus of endosymbiotic chlorophycean alga to alveolates [34], it now seems more likely that *cox2* split and relocated to the nuclear genome independently in both Alveolata and Chlorophyceae [47,48].

#### 2.1.2. Mitochondrial Cox2 Fission in Wasps Campsomeris (Arthropoda: Insecta)

*Cox2* has also been recently shown to be split into two complementary genes in the mtDNA of scoliid wasps (Hymenoptera: Scoliidae) of the genus *Campsomeris* (*Dielis*) [43]. This fission is unique in the sense that it is the only known case of a fragmented mitochondrial protein-coding gene in animals. It occurred relatively recently on the evolutionary scale, following separation of the *Campsomeris* lineage, and is not found in related hymenopterans, including *Scolia* (Scoliidae), *Myzinum* (Tiphiidae), and others. The fission was likely caused by insertion of a 3 kb non-mitochondrial DNA element bearing several genes, thus selecting against the general trend of mitogenome streamlining. The division of *cox2* in *Campsomeris* does not imply that it is a predecessor of piecewise gene transfer to the nucleus. However, it may favour eventual *cox2* transfer. Interestingly, preliminary comparative analyses of mitochondrial and total *Campsomeris* proteomes using antibodies specific for COXIIA and COXIIB revealed that all, or a portion of *cox2b* might have been copied to the nuclear genome and expressed there as a part of a chimeric protein, possibly resulting from fusion with a nuclear exonic sequence. This scenario is the more likely since, despite certain differences between the standard and invertebrate mitochondrial genetic codes (e.g., TGA specify Trp in invertebrate mtDNA but a translation stop in the nucleus), the entire *cox2b* could be translated on cytosolic ribosomes into the full-length polypeptide without a need for codon modification through a base substitution or for RNA editing. If the identity of the putative chimeric COXIIB is confirmed by direct protein sequencing, the fusion protein might represent either an evolving new protein, or an early stage of the acquisition of regulatory sequences by partially transferred mitochondrial *cox2*.

### 2.2. Transfer to the Nucleus of rpl2 in Eudicots (Tracheophyta: Angiospermae)

The *rpl2* gene represents another example of a split mitochondrial gene with intermediate stages of its transfer to the nucleus preserved in some lineages. RPL2 is a ribosomal protein functioning in the mitochondrial matrix in land plants and protists *Reclinomonas* (Excavata: Jacobea). The protein is relatively hydrophilic with the grand average of hydropathy (GRAVY) score for rice (*Oryza*) RPL2 equaling −0.43, thus being much lower than those of transmembrane-anchored COXI or COXII (0.69 and 0.19, respectively). It has been proposed that part of *rpl2*, located between the intron and 3’ end of the gene, was transferred to the nuclear genome in the ancestor of core eudicots, most likely preceding the generation of a stop codon TAA that left mitochondrial *rpl2* shortened to its 5’ section [49]. Following fission, the 5’ section of *rpl2* was transferred to the nucleus in legumes, lettuce and probably several other eudicot lineages [1,49]. Although it seems that the 3’ *rpl2* has never functioned in plant mtDNA as a separate gene, mitochondrial 3’ *rpl2* pseudogenes have been widely retained in eudicots for a relatively long time. It is rather unusual for mtDNA-encoded sequences to not be eliminated following their downgrade to pseudogene status, and suggests acquisition by them or their transcripts of some regulatory function(s).

In maize and probably also wheat, the entire mitochondrial *rpl2* has been relocated to the nucleus. Nuclear intact and 3’ *rpl2* genes, as well as the nuclear 5’ *rpl2* of lettuce, but not cotton, tomato or *Arabidopsis*, acquired mitochondrion-targeting presequences. It has been suggested that some nucleus-encoded RPL2s utilize internal mitochondrion-targeting sequences during their transfer to the mitochondrial matrix. The sole intron that is present in mtDNA-encoded full-length *rpl2* is preserved in mitochondrial 5’ *rpl2* (nuclear 5’ *rpl2* is intronless).

### 2.3. Nuclear Relocation of the 3’ End-Fragment of cox1 in the Majority of Protists

*Cox1* is the most often split mitochondrial gene in protists. It encodes subunit I of COX. Structurally, typical COXI is dominated by a 12-helix transmembrane pore-forming domain, always encoded by mtDNA. The binuclear heme a_3_-Cu_B_ active site, where molecular oxygen is reduced to water, is located in pore B and accepts electrons from heme a. Short N- and C-terminal regions of COXI are exposed on the mitochondrial matrix side. The split *cox1* has been described in many taxa of Protozoa and Chromista, but has not been found in plants, fungi, or animals [50]. In all identified fragmentation cases, the small 3’ region of *cox1* (*cox1-c*), encoding an on average 25-amino acid-long C-terminus of COXI, has been functionally transferred to the nucleus and is no longer recognizable in mtDNA either as a separate entity or a part of *cox1*. The COXI domain, encoded by *cox1-c*, contains a consensus motif SPPPXH with a conserved His residue that is proposed to be involved in the control of proton entrance into oxygen reduction pathway. The *cox1* split has punctate distribution across several major eukaryotic groups (Figure 1), suggesting that *cox1-c* was transferred to the nucleus early in eukaryotic evolution or was transferred many times independently.

### 2.4. Transfer of sdhB in Euglenozoa

In contrast to all other identified fragmented genes of mitochondrial origin, *sdhB* is the only gene, with a genes-in-pieces arrangement, known to reside exclusively in the nuclear genome. The gene encodes an electron transfer-mediating iron-sulfur subunit of tetrameric succinate dehydrogenase (Complex II of the respiratory chain, localized to the inner mitochondrial membrane from the matrix side). SDHB is a hydrophilic protein with a GRAVY score for the human subunit equalling −0.41. In eukaryotes, *sdhB* is usually present in the nuclear genome, with the exception of red algae and jakobid flagellates, in which it is encoded by mtDNA. The *sdhB* gene, split into *sdhB-n* and *sdhB-c*, exists in the nuclear genome of free-living *Euglena* and the trypanosomatids *Trypanosoma* and *Leishmania* [51]. No mtDNA-encoded *sdhB* gene has been identified in *Diplonema*, and given the phylogenetic closeness of diplonemids and trypanosomatids or euglenids, this gene is likely split into derived genes in the nuclear genome of diplonemids as well. Since no case of a partial nuclear transfer of *sdhB* is known, it is unclear whether *sdhB* fission occurred in mitochondria, during transfer or following transfer of an intact gene to the nuclear genome. As in the case of *cox2*, the split of *sdhB* in euglenozoons occurred within a relatively less conserved part of the gene that is apparently tolerant of such a disruption, between regions specifying the Fer2 and Fer4 domains.

## 3. Fragmented RNA Genes

Fragmented and sometimes rearranged rRNA genes have been found in bacterial, archaeal, nuclear, mitochondrial and chloroplast genomes. Small subunit (SSU) and/or large subunit (LSU) rRNA gene fragmentation and/or rearrangement were found in mtDNA of the fungus *Halorophidium* [55], ciliates *Tetrahymena* and *Paramecium* [37,56], green algae (Chlorophyta) [57,58,59], apicomplexans *Theleria* and *Plasmodium*, dinoflagellates *Oxyrrhus* and *Alexandrium* [60,61,62,63], Placozoa [64,65] and mollusks *Crassostrea* [66,67,68,69]. Interestingly, not all sequences presumed to be necessary for functional rRNAs have been identified, for example, in the mtDNA of *Plasmodium*, suggesting that some of them may be specified by derived genes relocated to the nucleus and posttranscriptionally imported to mitochondria. Likewise, only a small fragment of SSU rRNA has been identified in completely sequenced mtDNA of *Karlodinium* (Dinoflagellata) [70] suggesting that the remaining part of this rRNA may be encoded in the nuclear genome. A precedent for rRNA import to mitochondria exists in mammals where 5S rRNA is exclusively encoded in the nucleus and requires transfer to the mitochondria.

Split RNA genes are also known to encode fragmented tRNAs in Archaea [71,72,73]. Parts of a tRNA molecule can reassemble and function as an intact tRNA. In fact, the genomic tag hypothesis suggests that the top half of tRNA emerged earlier than the bottom half and that the two halves formed at some point functional RNA heteroduplex [22]. tRNA genes, coding for very truncated tRNAs that are missing their 3’ halves, have been identified in some arachnid lineages [74]. Those *trn* genes may have their complementary 3’ part transferred to the nucleus. An independent support for the partial tRNA gene relocation hypothesis comes from the fact that tRNA import to mitochondria occurs frequently throughout phylogeny, and in some cases, all tRNAs functioning in the mitochondria have to be imported from the nucleus. Nevertheless, other mechanisms, such as RNA editing using the 5’ portion of the acceptor stem as a template, may also explain the generation of functional tRNAs from their truncated genes [74].

## 4. Mechanisms of Piecewise Intercompartmental Gene Transfer

### 4.1. General Models

**Figure 2:**
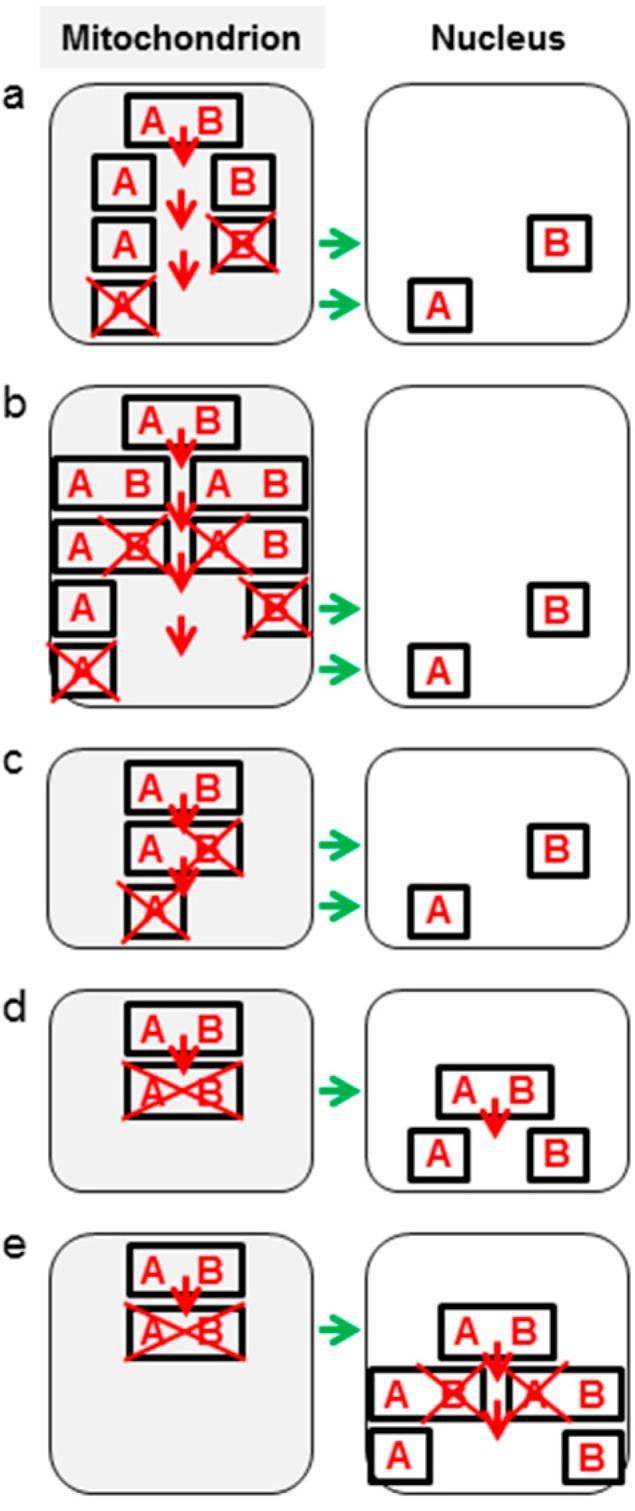
Schematic representation of different modes of mitochondrial gene fission and relocation to the nucleus. Letters A and B denote halves of the AB gene (a box). Cross-mark denotes pseudogenization or deletion of an indicated gene or its part. Green arrows represent intercompartmental gene transfer. (**a**,**b**) The AB gene split in mtDNA before its transfer to the nucleus. (**c**) Part B of the AB gene is copied to the nuclear genome without the fission of AB in the mitogenome. Subsequently, mitochondrial part B of the AB gene becomes pseudogenized and the part A of the gene relocates to the nucleus. (**d**,**e**) The AB gene is transferred to the nucleus as an intact gene and undergoes fission following integration into the nuclear genome.

Endosymbiotic relocation of genes in pieces can proceed according to three general models. In the first, an organelle gene is fragmented, most often into two complementary genes, followed by relocation to the nucleus of one or both of them (Figure 2a,b). Fission could result from conversion of an amino acid codon into a translational stop codon for one sequence and a start codon for the other, nucleotide substitution or frame-shift caused by an indel (Figure 2a), or gene duplication with subsequent selective loss of its parts leaving only complementary regions (Figure 2b).

The evolutionary process of functional gene fission was most recently demonstrated experimentally in *Escherichia coli* by insertional mutagenesis followed by a suppressor screen that identified composite *priA316::cat* split/rescue mutation [75]. Generation of this mutation involved an insertion of the *Cat^R^* gene that truncated PriA within the winged-helix domain (at the 154th codon), and an ACG(Thr)-to-ATG(Met) substitution that allowed reinitiation of translation at the 157th codon, so that *priA* was expressed in two functional pieces. Gene fission by insertion of a DNA fragment of unknown origin was the likely cause of the fragmentation of mitochondrial *cox2* in *Campsomeris* [43]. In this case, a putative homing nuclease QNU, encoded by the insert, might have been directly involved in gene splitting. In addition to *Campsomeris cox2*, other fragmented protein-coding, mtDNA-localized genes include *nad1*, *nad2* and *rps3* in ciliates (Alveolata) [37,38,39], and *ccmF* (*ccb*) orthologs of bacterial *ccl1* in land plants (liverwort *Marchantia* and angiosperms) [40,41,42]. It has been shown for the split of *cox2* in *Campsomeris* that both derived genes can be transcribed, processed into polyadenylated mRNAs and translated in the mitochondria [43]. Nevertheless, those cases of gene fission do not necessary represent migration-ready intermediates.

In a second model, the intercompartmental transfer is initiated by relocation of a copy of, for instance, the 3’-terminal region of a gene (Figure 2c). Subsequently, the mitochondrial 5’ open reading frame (ORF) is formed by a point mutation or an indel that creates a translational stop codon within the 3’-terminal region. In this model, mitochondrial gene fission is a consequence of partial gene copying to the nucleus.

In a third model, the gene fission follows transfer of a copy of an intact mitochondrial gene to the nucleus (Figure 2d,e).

### 4.2. Transfer and Integration of Nucleic Acids

In all models of endosymbiotic gene relocation, genes could be transferred between organelles and the nucleus through DNA and RNA intermediates. Experimental and bioinformatics studies in yeast, plants and other eukaryotes indicate that migration of mtDNA sequences to the nucleus is predominantly DNA mediated [2,5,9]. However, since transferred plant mitochondrial genes often resemble reverse transcribed mRNAs rather than the original mitochondrial genes containing introns [76,77], a modified mechanism has also been proposed with an additional step including generation of complementary DNA (cDNA) within mitochondria, followed either by its subsequent recombination with mitochondrial gene and transfer to the nucleus, or direct transfer to the nucleus [78].

Several possible routes for DNA escape from organelles have been identified. Disruptions of organelle membranes occur during autophagy, organelle fusion and fission, and other stress conditions; released organelle DNA could become accessible for uptake by the nuclear import pathways [79,80,81,82]. Direct association of the nucleus with mitochondria or chloroplasts and the uptake of whole mitochondria by nuclei [83,84,85,86,87] might also facilitate DNA exchange. In cases when organelles are maternally inherited, organelle-to-nucleus DNA transfer is thought to preferentially occur when DNA is released from degrading sperm mitochondria within the egg [3]. Lastly, viruses have been suggested as possible vectors in horizontal gene transfer to mitochondria, and they might also mediate transfer of mitochondrial genes to the nucleus. For instance, the acquisition of mitochondrial putative DNA repair gene, *MutS7*, by Octocorallia was proposed to occur through horizontal transfer, either from a nucleocytoplasmic large DNA virus or ɛ-proteobacterium [88]. Regarding mechanistic aspects of organellar DNA integration into the nuclear genome, sequencing of the insertion junctions in yeast, plants and human revealed the presence of 1-7 bp microhomologies that are indicative of DNA insertion by nonhomologous end-joining mechanism [89,90,91]. Insertions may also occur without the presence of microhomology as a result of blunt-end repair. In both cases, inserts often target open chromatin regions [90,91].

## 5. The Hydrophobicity-Importability Hypothesis

Depending on the physico-chemical characteristics of a protein encoded by a gene that is the subject of intercompartmental relocation, mitochondrial gene transfers might invoke the hydrophobicity hypothesis (or hypotheses) to explain both the feasibility of such transfers and their evolutionary preservation [1,92,93,94,95,96]. According to this hypothesis, hydrophobic regions, including transmembrane helices of proteins encoded by several mitochondrial genes, might prohibit those proteins from being effectively targeted back to mitochondria if they are translated on cytosolic ribosomes. They might be mistargeted to the endoplasmic reticulum (ER) [92,97], or they might be incorrectly translocated and assembled in the mitochondrial inner membrane [93,94]. This hypothesis was originally based on the comparison of the hydrophobicity of mitochondrial proteins encoded by nuclear and mitochondrial gene homologs showing that nuclear genome-encoded proteins were less hydrophobic than their mtDNA-encoded counterparts [76,92,98,99]. Further supporting this observation, the most hydrophobic mitochondrial proteins, apocytochrome b (COB) and COXI (or COXI(-)), have never been found to be functionally encoded within the nuclear genome. In addition, 12 of the 13 human mitogenome-encoded proteins, allotopically expressed in the cytoplasm of HeLa cells, even when fused with mitochondrial targeting presequence, were localized to the ER [100]. Only ATP8, which is the shortest of mtDNA-encoded proteins and contains a weakly hydrophobic transmembrane domain, was targeted to the mitochondria.

Direct experimental verification of the hydrophobicity hypothesis came from studies of *cox2* in legumes [99] and yeast [101]. Legumes contain both a mitochondrial and nuclear copy of *cox2*. COXII polypeptides encoded in these two cellular compartments display similar hydropathicity profiles, except for a decrease in the hydrophobicity of the first transmembrane helix of nucleus-encoded COXII. In vitro assays were conducted to determine the significance of this difference for the mitochondrial import of COXII translated in the cytosol [99]. The import of [^35^S]methionine-labelled mtDNA and nuclear genome encoded COXII to mitochondria was assessed by the appearance of an additional protein band in the presence of isolated mitochondria that was protected from added protease, and depended on the preservation of the mitochondrial inner membrane potential. The intramitochondrial location of imported proteins was queried by rupturing the outer mitochondrial membrane by osmotic shock, allowing the externally added protease to gain access to the intermembrane space, but not the mitochondrial matrix. Those experiments showed that mtDNA-encoded COXII could not be transported into mitochondria, even when fused with a mitochondrial targeting sequence that supports import of the nucleus-encoded COXII. However, removal of the first transmembrane helix from the mtDNA-specified COXII promoted its import. Moreover, they showed that the first transmembrane helix is not only inhibitory to the import, but it cannot pass the inner mitochondrial membrane, thus preventing COXII from reaching the correct topology required for the assembly and function of the respiratory Complex IV. Importantly, the change by site-directed mutagenesis of the two hydrophobic Leu residues, that contributed most to the difference in hydrophobicity between the first transmembrane helix of the mitochondrion and nucleus-encoded COXII, to polar Glu and neutral Gly also promoted import of mtDNA-encoded COXII. This finding was independently supported by the suppression of the mitochondrial import of nucleus-encoded COXII following introduction of the reciprocal amino acid changes (from Glu and Gly to Leu) into its first transmembrane helix. The in vitro studies using isolated mitochondria of legumes were further supported by in vivo experiments with yeast COXII [101]. Nuclear-recoded *Saccharomyces cerevisiae* COXII fused to mitochondrial targeting sequence failed to complement growth defects of yeast *cox2*-60 strain containing deletion in mitochondrial *cox2*. On the other hand, random mutagenesis-derived replacement of hydrophobic Trp of the first transmembrane domain of allotopically expressed COXII with positively charged Arg supported growth of *cox2*-60 strain on a nonfermentable carbon source, and partially restored activity of COX in *cox2*-60. In aggregate, these experiments demonstrated that decreasing the hydrophobicity of the first transmembrane helix of COXII is essential for the import into the mitochondria of COXII that has been translated on cytosolic ribosomes.

Splitting mitochondrial genes into derived genes, coding for hydrophobic and hydrophilic parts of a protein, might increase the probability of functional nuclear transfer at least for the segment of a gene encoding a more hydrophilic domain. Such a scenario is supported by the exclusive relocation to the nucleus of *cox1-c* encoding matrix-exposed hydrophilic C-terminal domain of COXI [50], or nuclear relocation of *cox2b* encoding intermembrane space hydrophilic domain of COXII that, in some cases, was the only part of *cox2* transferred to the nucleus [33,34,46], and in other cases likely preceded relocation of *cox2a* (there are no known cases of *cox1(-)* or *cox2a* encoded in the nucleus with, respectively, functional *cox1-c* or *cox2b* still residing in mtDNA). Moreover, mitochondrial gene fission coupled with nuclear relocation of a portion of a gene encoding a less hydrophobic protein domain might allow for a compromise between apparently counteracting processes of organellar genome streamlining and retention of some organellar genes. Retention of certain genes may have a selective advantage by allowing redox-regulated expression of respiratory complex proteins directly in the organelle (the collocalization for redox regulation hypothesis [96,102]), and partial gene transfer to the nucleus would not abolish organelle control of the activities of protein complexes specified by those genes.

Importantly, since the introduction of foreign genes into animal mitochondria remains challenging, the incorporation of fragmented mitochondrial genes into the nuclear genome might be a better strategy in the development of gene therapies for mitochondrial disorders. It might be easier for the products of allotopically expressed fragmented versus intact mitochondrial genes to be targeted to the mitochondria, resume there their intended activities, and functionally replace inactive or missing products of mutated mitochondrial genes.

Other conditions would likely also have to occur to promote evolutionary preservation of long-term nuclear relocation of a mitochondrial gene, including for instance, lower mutation rates in the nuclear genome compared to that of mtDNA. Nuclear genomes of plants have higher mutation rates compared to organellar genomes, and there are known cases of *cox2* wherein the gene is not only expressed from both nuclear and mitochondrial genomes, but also has been apparently lost from the nucleus in some groups following the establishment of its expression there [98]. In addition, interactions between mitochondrial and cytosolic proteins may interfere with the import of nucleus-encoded mitochondrial proteins [97]. The hydrophobicity hypothesis alone cannot, for instance, explain why the *rps10* gene, encoding a relatively hydrophilic ribosomal protein, has been functionally transferred to the nuclear genome late in the mitochondrial evolution of angiosperms but not in other plants, even though it can be imported without mitochondrial targeting presequence [77].

## 6. Partial Gene Conversion

The coexistence of multicompartmental homologs of a given gene in a single cell following transfer of an organellar gene to the nucleus, or because of endosymbiosis, may result in gene conversion. This process is distinct from intercompartmental gene transfer *sensu stricto* because it involves genes already present in both involved cellular compartments. During gene conversion, part of a native nuclear or organellar gene is replaced by the corresponding part of its homolog copied from other organellar genome or from an endosymbiotic species. Gene conversion might occur through integration of a fragment of genomic DNA or cDNA, followed by homologous or homeologous recombination between the two organellar or nuclear paralogs [103]. Alternatively, the converting genomic DNA fragment or cDNA could recombine directly with their target sequences [104]. In all cases described to date, converted genes exist in a form of continuous entity. Nevertheless, the process of gene conversion can also potentially lead to gene truncation or fission if the recombination was imprecise or involves a pseudogene. Owing to difficulties in its detection, there are very few cases of gene conversion known to date, but the process might be more common than is currently appreciated. In fact, plastid gene conversion has been experimentally demonstrated to be an efficient mutation-correcting mechanism that keeps mutation rates in chloroplast genomes at lower levels than those determined in nuclear genomes [105].

### 6.1. Chimeric Mitochondrial Genes

Intermitochondrial interspecies gene conversion has been identified for *rps11* and *atp1* genes in angiosperms. The 5’ half of the *rps11* gene of *Sanguinaria* (Papaveraceae) is of native, basal eudicot origin, whereas its 3’ half is of monocot origin [104]. The horizontal transfer of *rps11* from a monocot to *Sanguinaria* is evolutionarily young since other analyzed species of Papaveraceae contained only non-chimeric *rps11* genes. The other case of gene conversion is represented by the *atp1* gene of a parasitic flowering plant *Pilostyles thurberi* (Cucurbitales), centrally-located region II of which has been replaced multiple times and independently by orthologous sequences from the fabalean host plant, *Psorothamnus* or *Dalea* in Arizona and Texas isolates, respectively [106]. Interestingly, the mitochondrial *atp1* gene of several lamiales also underwent short-patch conversion by a homologous *atpA* of chloroplast origin (but not vice versa) [107]. Both *rps11* and *atp1* are represented by a single gene copy in the mtDNA; hence, their chimeric structures likely result from recombination between native alleles and transiently present foreign DNA [103,107].

### 6.2. Mitochondrial-to-Nuclear Gene Conversion

Simultaneous existence of nuclear and mitochondrial gene homologs has been shown for *cox2* in some legumes, *rpl5* in grasses, and *sdh4* in *Populus* [78,98,108,109]. Specifically, the mitochondrial ribosomal protein gene *rpl5* has been transferred to the nucleus at least three times in grasses, and in many of those cases, transcribed copies of *rpl5* are present both in mtDNA and the nuclear genome. One of the consequences of such gene arrangements is *rpl5* chimerism found in the nuclear genomes of *Lolium* and *Gynerium* [78], where a 0.1 kb portion of a nuclear *rpl5* gene has been replaced by a corresponding sequence of its mitochondrial counterpart.

## 7. Concluding Remarks

Both nuclear relocation of organellar genes and their fission into complementary genes may, in the long term, carry a selective advantage by increasing organismal fitness. However, whether and in what circumstances the gene fission and intercompartmental transfer might be mechanistically linked has been a subject of controversy, and apparently depends on the physicochemical properties of a relocated gene product, the subcellular localization of its activity, and the mechanism of gene split and relocation. In cases when only a portion of an organellar gene is copied to the nucleus without prior gene fission or partial duplication, gene fragmentation constitutes an integral part of partial gene translocation. Otherwise, both gene transfer and fission represent stochastic events that can be initiated independently of each other. Nevertheless, in the cellular realm, mutual interference of gene transfer and fission seems unavoidable and may contribute to the evolutionary preservation of both. Perhaps the best characterized paradigm in this context is a situation wherein a gene split increases the probability of functional transfer to the nucleus for a part of the gene encoding hydrophilic domains of an otherwise largely hydrophobic protein. The shorter and hydrophilic fragment would be easier to import back to the mitochondria than an intact protein and could resume activity there as a heterodimer with mtDNA-encoded complementary hydrophobic domains. Of importance in this context, facilitation of the intercompartmental transfer by gene fission may have implications for the development of therapeutic applications based on the allotopic expression of mitochondrial genes. On the other hand, intercompartmental relocation of a fragmented gene may also facilitate long-term preservation of the split-gene arrangement, especially in situations wherein the nuclear genome has a lower mutation rate than the organellar genome, and the organellar homolog of a nuclear gene has become a pseudogene or been lost by other means.

## Figures and Tables

**Table 1:** Mitochondrial proteins encoded by genes split into complementary genes. The taxonomy follows revised classification of extant organisms proposed in [36].

Heterodimeric or Trimeric Protein	Gene		Taxonomy	Reference
	Mitochondrial	Nuclear		
NAD1	*nad1a*, *nad1b*	-	*Paramecium* and other ciliates (Chromista: Alveolata)	[37,38,39]
NAD2	*nad2a*, *nad2b*	-		
RPS3	*rps3a*, *rps3b*	-		
CCMF	*ccmF_N_*, *ccmF_C1_*, *ccmF_C2_*	-	liverwort *Marchantia* (Plantae: Marchantiophyta)	[40,41,42]
	*ccmF_N_* (=*ccb577*), *ccmF_C_*	-	*Triticum* and most of the land plants (Plantae: Tracheophyta: Angiospermae)	
	*ccmF_N1_*, *ccmF_N2_*, *ccmF_C_* (=*ccb452*)	-	*Brassica*, *Arabidopsis* (Plantae: Tracheophyta: Angiospermae: Brassicaceae)	
COXII	*cox2a*, *cox2b*	-	*Campsomeris* (Animalia: Arthropoda: Insecta)	[43]
	*cox2a*	*cox2b*	*Scenedesmus* and other Sphaeropleales, Chaetophorales (Plantae: Chlorophyta: Chlorophyceae)	
	-	*cox2a*, *cox2b*	*Chlamydomonas* and other Chlamydomonadales (Plantae: Chlorophyta: Chlorophyceae); *Plasmodium* and other apicomplexan parasites, *Karlodinium* and other dinoflagellates, *Perkinsus* (Chromista: Alveolata)	[33,34,35,44,45,46,47,48]
RPL2	*5’rpl2*	*3’rpl2*	tomato, *Arabidopsis*, cotton (Plantae: Angiospermae: Eudicotidae)	[1,49]
	-	*5’rpl2*, *3’rpl2*	legumes, lettuce (Plantae: Angiospermae: Eudicotidae)	
COXI	*cox1(-)*	*cox1-c* (=*Dictiostelium coxIV*)	*Trypanosoma*, *Euglena* (Protozoa: Euglenozoa); *Malawimonas* (Protozoa: Loucozoa); *Acanthamoeba*, *Dictiostelium*, *Physarum* (Protozoa: Amoebozoa); *Phytophtora*, *Thalassiosira* (Chromista: Stramenopiles); *Plasmodium*, *Tetrahymena* (Chromista: Alveolata); *Bigielowiella* (Chromista: Rhizaria); *Emiliania* (Chromista: Hacrobia)	[50]
SDHB	-	*sdhb-n*, *sdhB-c*	*Diplonema*, *Trypanosoma*, *Euglena* (Protozoa: Euglenozoa)	[51]

## References

[1] Adams K.L., Palmer J.D. (2003). Evolution of mitochondrial gene content: gene loss and transfer to the nucleus. Mol. Phylogenet. Evol..

[2] Timmis J.N., Ayliffe M.A., Huang C.Y., Martin W. (2004). Endosymbiotic gene transfer: organellar genomes forge eukaryotic chromosomes. Nat. Rev. Genet..

[3] Leister D. (2005). Origin, evolution and genetic effects of nuclear insertions of organellar DNA. Trends Genet..

[4] Keeling P.J., Palmer J.D. (2008). Horizontal gene transfer in eukaryotic evolution. Nat. Rev. Genet..

[5] Kleine T., Maier U.G., Leister D. (2009). DNA transfer from organellars to the nucleus: The idiosyncratic genetics of endosymbiosis. Annu. Rev. Plant. Biol..

[6] Bock R. (2017). Witnessing genome evolution: Experimental reconstruction of endosymbiotic and horizontal gene transfer. Annu. Rev. Genet..

[7] Ku C., Nelson-Sathi S., Roettger M., Sousa F.L., Lockhart P.J., Bryant D., Hazkani-Covo E., McInerney J.O., Landan G., Martin W.F. (2015). Endosymbiotic origin and differential loss of eukaryotic genes. Nature.

[8] Harish A., Kurland C.G. (2017). Mitochondria are not captive bacteria. J. Theor. Biol..

[9] Bock R., Timmis J.N. (2008). Reconstructing evolution: Gene transfer from plastids to the nucleus. Bioessays.

[10] Bensasson D., Zhang D., Hartl D.L., Hewitt G.M. (2001). Mitochondrial pseudogenes: Evolution’s misplaced witnesses. Trends Ecol. Evol..

[11] Hazkani-Covo E., Zeller R.M., Martin W. (2010). Molecular poltergeists: Mitochondrial DNA copies (NUMTs) in sequenced nuclear genomes. PLoS Genet..

[12] Ju Y.S. (2016). Intracellular mitochondrial DNA transfers to the nucleus in human cancer cells. Curr. Opin. Genet. Dev..

[13] Noutsos C., Kleine T., Armbruster U., DalCorso G., Leister D. (2007). Nuclear insertions of organellar DNA can create novel patches of functional exon sequences. Trends Genet..

[14] Muller H.J. (1964). The relation of recombination to mutational advance. Mutat. Res..

[15] Lynch M. (1996). Mutation accumulation in transfer RNAs: Molecular evidence for Muller’s rachet in mitochondrial genomes. Mol. Biol. Evol..

[16] Berg O.G., Kurland C.G. (2000). Why mitochondrial genes are most often found in nuclei. Mol. Biol. Evol..

[17] Allen J.F., Raven J.A. (1996). Free-radical-induced mutation vs redox regulation: Costs and benefits of genes in organellars. J. Mol. Evol..

[18] Selosse M., Albert B., Godelle B. (2001). Reducing the genome size of organellars favours gene transfer to the nucleus. Trends Ecol. Evol..

[19] Gemmell N.J., Braischer T.L. (2001). Organellar genome evolution. Trends Ecol. Evol..

[20] Gray M.W., Schnare M.N., Zimmermann R., Dahlberg A. (1996). Evolution of rRNA gene organization. Ribosomal RNA: Structure, Evolution, Processing, and Function in Protein Biosynthesis.

[21] Evguenieva-Hackenberg E. (2005). Bacterial ribosomal RNA in pieces. Mol. Microbiol..

[22] Kanai A. (2015). Disrupted tRNA Genes and tRNA fragments: A perspective on tRNA gene evolution. Life.

[23] Shiba K., Schimmel P. (1992). Functional assembly of a randomly cleaved protein. Proc. Natl. Acad. Sci. USA.

[24] Magliery T.J., Wilson C.G., Pan W., Mishler D., Ghosh I., Hamilton A.D., Regan L. (2005). Detecting protein-protein interactions with a green fluorescent protein fragment reassembly trap: scope and mechanism. J. Am. Chem. Soc..

[25] Fukutani Y., Ishii J., Kondo A., Ozawa T., Matsunami H., Yohda M. (2017). Split luciferase complementation assay for the analysis of G protein-coupled receptor ligand response in *Saccharomyces cerevisiae*. Biotechnol. Bioeng..

[26] Dolan M.J., Luan H., Shropshire W.C., Sutcliffe B., Cocanougher B., Scott R.L., Frechter S., Zlatic M., Jefferis G.S.X.E., White B.H. (2017). Facilitating neuron-specific genetic manipulations in *Drosophila melanogaster* using a split GAL4 repressor. Genetics.

[27] Kaya H., Ishibashi K., Toki S. (2017). A split *Staphylococcus aureus* Cas9 as a compact genome-editing tool in plants. Plant. Cell Physiol..

[28] Snel B., Bork P., Huynen M. (2000). Genome evolution. Gene fusion versus gene fission. Trends Genet..

[29] Kummerfeld S.K., Teichmann S.A. (2005). Relative rates of gene fusion and fission in multi-domain proteins. Trends Genet..

[30] Marianayagam N.J., Sunde M., Matthews J.M. (2004). The power of two: Protein dimerization in biology. Trends Biochem. Sci..

[31] Hashimoto K., Nishi H., Bryant S., Panchenko A.R. (2011). Caught in self-interaction: evolutionary and functional mechanisms of protein homooligomerization. Phys. Biol..

[32] Lynch M. (2012). The evolution of multimeric protein assemblages. Mol. Biol. Evol..

[33] Kück U., Jekosch K., Holzamer P. (2000). DNA sequence analysis of the complete mitochondrial genome of the green alga *Scenedesmus obliquus*: Evidence for UAG being a leucine and UCA being a non-sense codon. Gene.

[34] Nedelcu A.M., Lee R.W., Lemieux C., Gray M.W., Burger G. (2000). The complete mitochondrial DNA sequence of *Scenedesmus obliquus* reflects an intermediate stage in the evolution of the green algal mitochondrial genome. Genome Res..

[35] Pérez-Martínez X., Antaramian A., Vazquez-Acevedo M., Funes S., Tolkunova E., d’Alayer J., Claros M.G., Davidson E., King M.P., González-Halphen D. (2001). Subunit II of cytochrome c oxidase in *Chlamydomonad* algae is a heterodimer encoded by two independent nuclear genes. J. Biol. Chem..

[36] Ruggiero M.A., Gordon D.P., Orrell T.M., Bailly N., Bourgoin T., Brusca R.C., Cavalier-Smith T., Guiry M.D., Kirk P.M. (2015). A higher level classification of all living organisms. PLoS ONE.

[37] Burger G., Zhu Y., Littlejohn T.G., Greenwood S.J., Schnare M.N., Lang B.F., Gray M.W. (2000). Complete sequence of the mitochondrial genome of *Tetrahymena pyriformis* and comparison with *Paramecium aurelia* mitochondrial DNA. J. Mol. Biol..

[38] Edqvist J., Burger G., Gray M.W. (2000). Expression of mitochondrial protein-coding genes in *Tetrahymena pyriformis*. J. Mol. Biol..

[39] Swart E.C., Nowacki M., Shum J., Stiles H., Higgins B.P., Doak T.G., Schotanus K., Magrini V.J., Minx P., Mardis E.R. (2012). The *Oxytricha trifallax* mitochondrial genome. Genome Biol. Evol..

[40] Handa H., Bonnard G., Grienenberger J.M. (1996). The rapeseed mitochondrial gene encoding a homologue of the bacterial protein Ccl1 is divided into two independently transcribed reading frames. Mol. Gen. Genet..

[41] Unseld M., Marienfeld J.R., Brandt P., Brennicke A. (1997). The mitochondrial genome of *Arabidopsis thaliana* contains 57 genes in 366,924 nucleotides. Nat. Genet..

[42] Rayapuram N., Hagenmuller J., Grienenberger J.M., Bonnard G., Giegé P. (2008). The three mitochondrial encoded CcmF proteins form a complex that interacts with CCMH and c-type apocytochromes in *Arabidopsis*. J. Biol. Chem..

[43] Szafranski P. (2017). Evolutionarily recent, insertional fission of mitochondrial *cox2* into complementary genes in bilaterian Metazoa. BMC Genom..

[44] Funes S., Davidson E., Reyes-Prieto A., Magallón S., Herion P., King M.P., González-Halphen D. (2002). A green algal apicoplast ancestor. Science.

[45] Gardner M.J., Hall N., Fung E., White O., Berriman M., Hyman R.W., Carlton J.M., Pain A., Nelson K.E., Bowman S. (2002). Genome sequence of the human malaria parasite *Plasmodium falciparum*. Nature.

[46] Rodríguez-Salinas E., Riveros-Rosas H., Li Z., Fucíková K., Brand J.J., Lewis L.A., González-Halphen D. (2012). Lineage-specific fragmentation and nuclear relocation of the mitochondrial *cox2* gene in chlorophycean green algae (Chlorophyta). Mol. Phylogenet. Evol..

[47] Waller R.F., Keeling P.J., van Dooren G.G., McFadden G.I. (2003). Comment on ´A green algal apicoplast ancestor´. Science.

[48] Waller R.F., Keeling P.J. (2006). Alveolate and chlorophycean mitochondrial *cox2* genes split twice independently. Gene.

[49] Adams K.L., Ong H.C., Palmer J.D. (2001). Mitochondrial gene transfer in pieces: Fission of the ribosomal protein gene *rpl2* and partial or complete gene transfer to the nucleus. Mol. Biol. Evol..

[50] Gawryluk R.M., Gray M.W. (2010). An ancient fission of mitochondrial *cox1*. Mol. Biol. Evol..

[51] Gawryluk R.M., Gray M.W. (2009). A split and rearranged nuclear gene encoding the iron-sulfur subunit of mitochondrial succinate dehydrogenase in Euglenozoa. BMC Res. Notes.

[52] Adl S.M., Simpson A.G., Lane C.E., Lukeš J., Bass D., Bowser S.S., Brown M.W., Burki F., Dunthorn M., Hampl V. (2012). The revised classification of eukaryotes. J. Eukaryot. Microbiol..

[53] Merchant S.S., Prochnik S.E., Vallon O., Harris E.H., Karpowicz S.J., Witman G.B., Terry A., Salamov A., Fritz-Laylin L.K., Maréchal-Drouard L. (2007). The *Chlamydomonas* genome reveals the evolution of key animal and plant functions. Science.

[54] Prochnik S.E., Umen J., Nedelcu A.M., Hallmann A., Miller S.M., Nishii I., Ferris P., Kuo A., Mitros T., Fritz-Laylin L.K. (2010). Genomic analysis of organismal complexity in the multicellular green alga *Volvox carteri*. Science..

[55] Forget L., Ustinova J., Wang Z., Huss V.A., Lang B.F. (2002). *Hyaloraphidium curvatum*: A linear mitochondrial genome, tRNA editing, and an evolutionary link to lower fungi. Mol. Biol. Evol..

[56] Heinonen T.Y., Schnare M.N., Young P.G., Gray M.W. (1987). Rearranged coding segments, separated by a transfer RNA gene, specify the two parts of a discontinuous large subunit ribosomal RNA in *Tetrahymena pyriformis* mitochondria. J. Biol. Chem..

[57] Boer P.H., Gray M.W. (1988). Scrambled ribosomal RNA gene pieces in *Chlamydomonas reinhardtii* mitochondrial DNA. Cell.

[58] Denovan-Wright E.M., Lee R.W. (1994). Comparative structure and genomic organization of the discontinuous mitochondrial ribosomal RNA genes of *Chlamydomonas eugametos* and *Chlamydomonas reinhardtii*. J. Mol. Biol..

[59] Denovan-Wright E.M., Sankoff D., Spencer D.F., Lee R.W. (1996). Evolution of fragmented mitochondrial ribosomal RNA genes in *Chlamydomonas*. J. Mol. Evol..

[60] Kairo A., Fairlamb A.H., Gobright E., Nene V. (1994). A 7.1 kb linear DNA molecule of *Theileria parva* has scrambled rDNA sequences and open reading frames for mitochondrially encoded proteins. EMBO J..

[61] Feagin J.E., Gardner M.J., Williamson D.H., Wilson R.J. (1991). The putative mitochondrial genome of *Plasmodium falciparum*. J. Protozool..

[62] Slamovits C.H., Saldarriaga J.F., Larocque A., Keeling P.J. (2007). The highly reduced and fragmented mitochondrial genome of the early-branching dinoflagellate *Oxyrrhis marina* shares characteristics with both apicomplexan and dinoflagellate mitochondrial genomes. J. Mol. Biol..

[63] Kamikawa R., Inagaki Y., Sako Y. (2007). Fragmentation of mitochondrial large subunit rRNA in the dinoflagellate *Alexandrium catenella* and the evolution of rRNA structure in alveolate mitochondria. Protist.

[64] Dellaporta S.L., Xu A., Sagasser S., Jakob W., Moreno M.A., Buss L.W., Schierwater B. (2006). Mitochondrial genome of *Trichoplax adhaerens* supports placozoa as the basal lower metazoan phylum. Proc. Natl. Acad. Sci. USA.

[65] Signorovitch A.Y., Buss L.W., Dellaporta S.L. (2007). Comparative genomics of large mitochondria in placozoans. PLoS Genet..

[66] Milbury C.A., Gaffney P.M. (2005). Complete mitochondrial DNA sequence of the eastern oyster *Crassostrea virginica*. Mar. Biotechnol..

[67] Yu Z., Wei Z., Kong X., Shi W. (2008). Complete mitochondrial DNA sequence of oyster *Crassostrea hongkongensis*—A case of ‘Tandem duplication-random loss’ for genome rearrangement in *Crassostrea*?. BMC Genom..

[68] Ren J., Liu X., Zhang G., Liu B., Guo X. (2009). ´Tandem duplication-random loss´ is not a real feature of oyster mitochondrial genomes. BMC Genom..

[69] Milbury C.A., Lee J.C., Cannone J.J., Gaffney P.M., Gutell R.R. (2010). Fragmentation of the large subunit ribosomal RNA gene in oyster mitochondrial genomes. BMC Genom..

[70] Jackson C.J., Norman J.E., Schnare M.N., Gray M.W., Keeling P.J., Waller R.F. (2007). Broad genomic and transcriptional analysis reveals a highly derived genome in dinoflagellate mitochondria. BMC Biol..

[71] Randau L., Münch R., Hohn M.J., Jahn D., Söll D. (2005). *Nanoarchaeum equitans* creates functional tRNAs from separate genes for their 5’- and 3’-halves. Nature.

[72] Fujishima K., Sugahara J., Tomita M., Kanai A. (2008). Sequence evidence in the archaeal genomes that tRNAs emerged through the combination of ancestral genes as 5’ and 3’ tRNA halves. PLoS ONE.

[73] Chan P.P., Cozen A.E., Lowe T.M. (2011). Discovery of permuted and recently split transfer RNAs in Archaea. Genome Biol..

[74] Masta S.E., Boore J.L. (2008). Parallel evolution of truncated transfer RNA genes in arachnid mitochondrial genomes. Mol. Biol. Evol..

[75] Leroux M., Jani N., Sandler S.J. (2017). A *priA* mutant expressed in two pieces has almost full activity in *Escherichia coli* K-12. J. Bacteriol..

[76] Nugent J.M., Palmer J.D. (1991). RNA-mediated transfer of the gene *coxII* from the mitochondrion to the nucleus during flowering plant evolution. Cell.

[77] Adams K.L., Daley D.O., Qiu Y.L., Whelan J., Palmer J.D. (2000). Repeated, recent and diverse transfers of a mitochondrial gene to the nucleus in flowering plants. Nature.

[78] Wu Z., Sloan D.B., Brown C.W., Rosenblueth M., Palmer J.D., Ong H.C. (2017). Mitochondrial retroprocessing promoted functional transfers of *rpl5* to the nucleus in grasses. Mol. Biol. Evol..

[79] Thorsness P.E., White K.H., Fox T.D. (1993). Inactivation of *YME1*, a member of the ftsH-SEC18-PAS1-CDC48 family of putative ATPase-encoding genes, causes increased escape of DNA from mitochondria in *Saccharomyces cerevisiae*. Mol. Cell. Biol..

[80] Cerutti H., Jagendorf A.T. (1993). DNA strand-transfer activity in pea (*Pisum sativum* L.) chloroplasts. Plant Physiol..

[81] Campbell C.L., Thorsness P.E. (1998). Escape of mitochondrial DNA to the nucleus in *yme1* yeast is mediated by vacuolar-dependent turnover of abnormal mitochondrial compartments. J. Cell Sci..

[82] Thorsness P.E., Fox T.D. (1990). Escape of DNA from mitochondria to the nucleus in *Saccharomyces cerevisiae*. Nature.

[83] Thorsness P.E., Weber E.R. (1996). Escape and migration of nucleic acids between chloroplasts, mitochondria, and the nucleus. Int. Rev. Cytol..

[84] Brandes D., Schofield B.H., Anton E. (1965). Nuclear mitochondria?. Science.

[85] Yu H.S., Russell S.D. (1994). Occurrence of mitochondria in the nuclei of tobacco sperm cells. Plant Cell.

[86] Takemura G., Takatsu Y., Sakaguchi H., Fujiwara H. (1997). Intranuclear mitochondria in human myocardial cells. Pathol. Res. Pract..

[87] Bakeeva L.E., Skulachev V.P., Sudarikova Y.V., Tsyplenkova V.G. (2001). Mitochondria enter the nucleus (one further problem in chronic alcoholism). Biochemistry.

[88] Bilewitch J.P., Degnan S.M. (2011). A unique horizontal gene transfer event has provided the octocoral mitochondrial genome with an active mismatch repair gene that has potential for an unusual self-contained function. BMC Evol. Biol..

[89] Ricchetti M., Tekaia F., Dujon B. (2004). Continued colonization of the human genome by mitochondrial DNA. PLoS Biol..

[90] Lloyd A.H., Timmis J.N. (2011). The origin and characterization of new nuclear genes originating from a cytoplasmic organellar genome. Mol. Biol. Evol..

[91] Wang D., Timmis J.N. (2013). Cytoplasmic organellar DNA preferentially inserts into open chromatin. Genome Biol. Evol..

[92] Von Heijne G. (1986). Why mitochondria need a genome. FEBS Lett..

[93] Popot J.L., de Vitry C. (1990). On the microassembly of integral membrane proteins. Annu. Rev. Biophys. Biophys. Chem..

[94] Claros M.G., Perea J., Shu Y., Samatey F.A., Popot J.L., Jacq C. (1995). Limitations to in vivo import of hydrophobic proteins into yeast mitochondria. The case of a cytoplasmically synthesized apoapocytochrome b. Eur. J. Biochem..

[95] Daley D.O., Whelan J. (2005). Why genes persist in organelle genomes. Genome Biol..

[96] Johnston I.G., Williams B.P. (2016). Evolutionary inference across eukaryotes identifies specific pressures favoring mitochondrial gene retention. Cell Syst..

[97] Björkholm P., Ernst A.M., Hagström E., Andersson S.G. (2017). Why mitochondria need a genome revisited. FEBS Lett..

[98] Adams K.L., Song K., Roessler P.G., Nugent J.M., Doyle J.L., Doyle J.J., Palmer J.D. (1999). Intracellular gene transfer in action: Dual transcription and multiple silencings of nuclear and mitochondrial *cox2* genes in legumes. Proc. Natl. Acad. Sci. USA.

[99] Daley D.O., Clifton R., Whelan J. (2002). Intracellular gene transfer: Reduced hydrophobicity facilitates gene transfer for subunit 2 of cytochrome c oxidase. Proc. Natl. Acad. Sci. USA.

[100] Oca-Cossio J., Kenyon L., Hao H., Moraes C.T. (2003). Limitations of allotopic expression of mitochondrial genes in mammalian cells. Genetics.

[101] Supekova L., Supek F., Greer J.E., Schultz P.G. (2010). A single mutation in the first transmembrane domain of yeast COX2 enables its allotopic expression. Proc. Natl. Acad. Sci. USA.

[102] Allen J.F. (2015). Why chloroplasts and mitochondria retain their own genomes and genetic systems: Colocation for redox regulation of gene expression. Proc. Natl. Acad. Sci. USA.

[103] Hao W., Richardson A.O., Zheng Y., Palmer J.D. (2010). Gorgeous mosaic of mitochondrial genes created by horizontal transfer and gene conversion. Proc. Natl. Acad. Sci. USA.

[104] Bergthorsson U., Adams K.L., Thomason B., Palmer J.D. (2003). Widespread horizontal transfer of mitochondrial genes in flowering plants. Nature.

[105] Khakhlova O., Bock R. (2006). Elimination of deleterious mutations in plastid genomes by gene conversion. Plant J..

[106] Barkman T.J., McNeal J.R., Lim S.H., Coat G., Croom H.B., Young N.D., Depamphilis C.W. (2007). Mitochondrial DNA suggests at least 11 origins of parasitism in angiosperms and reveals genomic chimerism in parasitic plants. BMC Evol. Biol..

[107] Hao W., Palmer J.D. (2009). Fine-scale mergers of chloroplast and mitochondrial genes create functional, transcompartmentally chimeric mitochondrial genes. Proc. Natl. Acad. Sci. USA.

[108] Sandoval P., León G., Gómez I., Carmona R., Figueroa P., Holuigue L., Araya A., Jordana X. (2004). Transfer of *RPS14* and *RPL5* from the mitochondrion to the nucleus in grasses. Gene.

[109] Choi C., Liu Z., Adams K.L. (2006). Evolutionary transfers of mitochondrial genes to the nucleus in the *Populus* lineage and coexpression of nuclear and mitochondrial *Sdh4* genes. New Phytol..

